# Programmable Thermo-Responsive Self-Morphing Structures Design and Performance

**DOI:** 10.3390/ma15248775

**Published:** 2022-12-08

**Authors:** Surya Prakash Pandeya, Sheng Zou, Byeong-Min Roh, Xinyi Xiao

**Affiliations:** 1 Mechanical and Manufacturing Engineering Department, Miami University, Oxford, OH 45056, USA; 2 School of Transportation and Logistics Engineering, Wuhan University of Technology, Wuhan 430063, China; 3 School of Industrial and Systems Engineering, The University of Oklahoma, Norman, OK 73019, USA

**Keywords:** self-morphing, quantitative morphing analysis, thermos-responsive, autonomous design

## Abstract

Additive manufacturing (AM), also known as 3D printing, was introduced to design complicated structures/geometries that overcome the manufacturability limitations of traditional manufacturing processes. However, like any other manufacturing technique, AM also has its limitations, such as the need of support structures for overhangs, long build time etc. To overcome these limitations of 3D printing, 4D printing was introduced, which utilizes smart materials and processes to create shapeshifting structures with the external stimuli, such as temperature, humidity, magnetism, etc. The state-of-the-art 4D printing technology focuses on the “***form***” of the 4D prints through the multi-material variability. However, the quantitative morphing analysis is largely absent in the existing literature on 4D printing. In this research, the inherited material anisotropic behaviors from the AM processes are utilized to drive the morphing behaviors. In addition, the quantitative morphing analysis is performed for designing and controlling the shapeshifting. A material–process–performance 4D printing prediction framework has been developed through a novel dual-way multi-dimensional machine learning model. The morphing evaluation metrics, bending angle and curvature, are obtained and archived at 99% and 93.5% *R*^2^, respectively. Based on the proposed method, the material and production time consumption can be reduced by around 65–90%, which justifies that the proposed method can re-imagine the digital–physical production cycle.

## 1. Introduction

**Figure 1:**
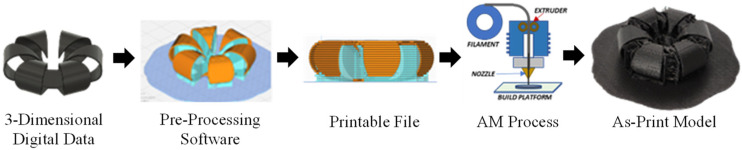
Traditional AM, 3D printing, workflow—from digital to physical.

Additive manufacturing (AM) is an easy-to-use technique that deposits materials layer-by-layer to create three-dimensional objects [1,2,3]. Compared with the traditional subtractive manufacturing processes, additive manufacturing provides more flexibility in the design, so that it can be widely used in the aerospace and automotive industries. However, in the digital–physical production cycle (shown in Figure 1), the as-built geometry always requires the use of support structures for the overhanging features. The final as-built 3D model in Figure 1 contains the model and the support volumes which need to be further post-processed. As a result, regardless of the benefits, such a layer-by-layer deposition method also requires additional post-processing steps, long building time, and poor surface finishes, specifically for the freeform surfaces.

To extend the use of 3D-printed objects, a fourth temporal dimension is added to 3D printing called 4D printing. With the additional dimension of time, 3D-printed objects are exposed to certain environmental stimulants, such as temperature, light, humidity, pH, and magnetism [4], to achieve the shape-morphing behavior. Due to its dynamic nature, 4D printing can be used in self-deployable systems, soft robotics, biomedical applications, etc.

Based on the variety of end-use applications, various smart materials [5], such as SMPs (Shape Memory Polymers), Hydrogels, and Magnetic nanoparticles, can be deployed on fused filament fabrication (FFF), stereolithography (SLA), and material jetting AM processes. The 3D-printed shapes are normally flat and simple, while they can be deformed to certain complex geometries due to the material behavior under different environmental stimuli. However, certain issues still hinder the major adoption of this technology: (1) lack of robustness in production; (2) the self-morphing process cannot be simulated and is unpredictable; and (3) only monolith shapeshifting is presented on whole 3D-printed objects. This paper investigates thermos-responsive self-morphing designs and performance using a novel machine learning model to measure the deformation quantitatively.

Smart materials that respond to external stimuli by changing shapes and sizes are among the most popular 4D printing applications. Among these smart materials, SMPs are the most widely used and easy to work with, as they can trigger shape change through heat or solvent stimuli. One of the examples is the water-responsive SMPs-hydrogels, which have been used as 4D printing materials when exposed to water. The other type of SMPs is thermo-responsive and can deform through heat/temperature. Besides the smart materials, the other widely used 4D technologies rely on multi-material printing by utilizing the different thermos-mechanical behaviors to achieve shapeshifting. In contrast, some other researchers [6] programmed the manufacturing patterns on a single material to achieve the anisotropic behavior for morphing. However, these 4D printing production approaches are based on a random design using trial and error methods, which lack robustness control. Furthermore, to analyze the deformation due to the various sources of the environmental simulants, some finite element analysis (FEA) [7] uses the material distribution per layer, but this process is costly and needs huge computational power and lacks control over the shape-change. Therefore, a deep understanding of the intersection of the process and the resultant performance for the self-morphing behavior is urgently needed. The developed process–performance framework can provide dual-way informing and guiding of the 3D print shapes and their associated deformation. A comprehensive digital-to-product 4D printing manufacturing process is presented in Figure 1.

**Figure 2:**
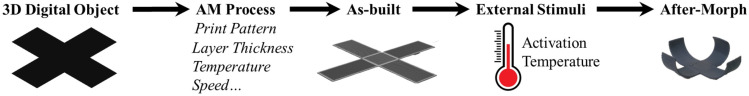
Thermo-responsive Self-morphing Design-Product Overall Workflow.

Figure 2 first presents a production workflow from a three-dimensional digital design to the physical 3D product through the traditional material deposition process. Specifically, the inherited anisotropic behavior of 3D-printed parts is utilized to achieve shape-morphing behaviors since the various printing patterns develop different strains on distinctive layers. The cross-layer strain variation ultimately causes the thermos-mechanical morphing matters. In this process, the critical aspects from the AM process parameters that will produce the structural anisotropic behaviors will reflect on the cross-layer strain level, which is the major driving factor of the morphing performance. Thus, a computational knowledge system that quantitatively measures the shape-morphing performance from the various process parameters is proposed in this paper.

**Figure 3:**
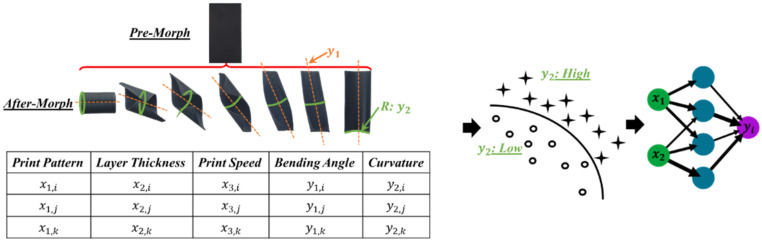
Overview of the proposed process–performance model.

Previous studies [8] have discovered that there exists a cause–effect relationship between the orientations of the AM hatch lines and the thermo-mechanical strain. In addition, the inverse-proportional qualitative study between the layer thickness and the strain has been investigated. In this research, a machine learning process–performance model has been developed to serve as a surrogate model to: (1) forwardly predict the freeform deformation from the defined process controlling variables and (2) backwardly guide the AM-ed three-dimensional digital model based on the desired after-morphing shapes. The overall insight into the proposed method is shown in Figure 3.

## 2. State-of-the-Art Review

There are various approaches used to achieve 4D printing. This section narrates the current thermos-responsive 4D printing mechanism and the anticipated morphing behaviors.

Zhang et al. [9] used thin, printed composite sheets made of paper and PLA, triggering shape transformation due to a difference in the coefficient of thermal expansion. They used a trial-and-error method for determining the self-morphing shapes. Kacergis et al. [10] constructed a composite bilayer structure consisting of PLA and TPU to achieve a self-bending hinge. It has been discovered that the printing speed is directly proportional to the strain developed, thus there is more deformation with an increase in printing speed. Tomec et al. [11] used wood-PLA (made by mixing different amounts of wood particles and PLA) with regular PLA to generate moisture-induced shape morphing. Using multi-material 3D printing technology, these composite structures can achieve a multistage deformation based on the combinations of two or three materials. Hence, a lower cost and robust real-life alternative is a challenge for this technology. Van Manen et al. [12] first presented a technique that used multiple design strategies to achieve complex 3D shapes, and varying thicknesses were integrated with the porosity of the constructs to program adjustable time delays for sequential shapeshifting. This simple and versatile method was able to deform the structures into the desired shape under stimuli. With a similar idea, Zheng et al. [13] used a hydrogel-based layered structure and designed a four-arm griper with considerable holding force. The printing direction was used to control the swelling direction of the hydrogel. Considering the influence of printing parameters, several FE methods were developed to control the deformation of the bilayer structure. Bodaghi et al. [14] investigated directly engineering the performance-driven functionality into materials with the help of printing parameters for 4D printing that affect the layer-by-layer programming process and shape-change, such as printing speed and liquefier temperature. They then used finite element (FE) formulation to generate functionally graded polymer beam strips that show self-coiling behavior in a controllable manner. Again, in 2019, Bodaghi et al. [15] used FDM printers to fabricate adaptive composite structures to achieve self-foldable structures using their thermomechanical properties and printing parameters. They investigated only the printing speed in detail, used the FE method to simulate the results, and created a self-coiling flower shape. Wang et al. [16] used FE analysis to predict the deformations of a monolayered SMP using the FE governing equations. They were able to preprogram self-folding origami structures using their simulation method. However, this method used monolayered structures and cannot be used to explain the effect of multiple layered prints with varying print parameters. Bouaziz et al. [17] conducted experimental tests to establish a constitutive Finite Element model to describe the thermomechanical cycle of a semi-crystalline shape memory polyurethane (SMPU) that can recover its shape after applying large strain during a shape memory cycle. The model was good at explaining hyper-elastic and time-dependent responses of both semi-crystalline and amorphous SMPs. However, due to material properties, the FE method can only be reliable for deformations. Various similar research was conducted for better-controlled deformation using FE models considering the influence of printing parameters, such as plate thickness [18], printing speed [19], and the printing direction [20]. These studies mostly focused on the effect of a single printing parameter to achieve shape morphing properties. A huge amount of computational power is needed for multiple variations of printing parameters, which is computationally inefficient. Zeng et al. [21] provided a method for programming the deformation of a bilayer structure with temperature as the actuating factor by studying three types of deformation behavior and creating a constitutive model using five printing parameters, namely, the print height, the print temperature, the filled form, and the stimulation temperature. Choi et al. [22] used dual layer SMPs to achieve 4D printing using materials that exhibit different expansion and found a method to achieve a perfect cylinder using a composite bilayer beam by ethanol absorption as a stimulant. Wang et al. [23] investigated the effect of nozzle temperature, filling angle, and geometric thickness on the shape memory properties of PLA. They found out that nozzle temperature and filling angle apparently affected shape recovery time and shape recovery force, respectively, while geometric thickness affected both recovery time and force simultaneously. Saad Y. et al. [24] found out that different bending deformations can be generated using patterns with shapes (circles, squares, hexagons, rhombuses, and triangles) of different sizes. These patterns of variable stiffness can be used in the actuators to control their degree of bending. They tested a total of five bio-inspired shapes. Rajkumar et al. [25] worked on quantifying and understanding the Fused Filament Fabrication (FFF) process parameters, mainly printing speed, print path, and infill density for three different printing materials, PLA, ABS, and HIPS. Bona Goo et al. [26] developed a 4D printing method using the anisotropic deformation of 3D-printed parts with thermal stimuli. A single thermoplastic filament (ABS) and a ME-type 3D printer were used. Transverse and longitudinal printing paths were used to program thermal anisotropy in a bidirectional manner to achieve 4D printing. Nezhad et al. [27] created a model to predict the morphing shape using a semi-empirical approach. They utilized printing speed, layer thickness, nozzle temperature, and raster angle as their process parameters. They only tested a few combinations of raster angles, and their proposed model is noncomprehensive in explaining the effect of raster angles on the deformation parameters. Song et al. [28] used the difference between the thermal expansion of bilayer structures that are mismatched due to the nature of the printing process and the print angles as the printing parameters. This mismatch in the coefficient of thermal expansion was then used to create a reduced bilayer plate model based on the coefficient of thermal expansion. This method could not fully explain more complex situations and could not relate simulation parameters with printing parameters. Other researchers have found that the process variant affects the as-built qualities [29,30,31,32,33,34,35,36,37,38,39]; they have indicated that the process plays a significant role in determining the anisotropic mechanical behaviors. However, there still lacks a linkage between such effects and morphing behaviors.

A multitude of research has been conducted on utilizing the printing parameters as the control factors for 4D prints. However, a review of the previous literature suggests that extensive research in constructing a simple constitutive model to explain the deformation behavior of a 3D-printed bilayer structure utilizing multiple printing parameters seems to be lacking.

## 3. Experimental Setup

The samples were 3D printed using a fused filament fabrication (FFF) process (Dremel 3D45; Dremel Manufacturing Co., USA), and Eco-ABS (Acrylonitrile Butadiene Styrene) filaments (Diameter = 1.75 mm) were adopted as the printing filament. The mechanical and thermal properties of Eco-ABS are shown in Table 1.

Having an enclosed printing chamber with a constant temperature, the bed temperature and nozzle temperature of the 3D printer were set to 60 and 230 °C, respectively. Generally, for extrusion-based printers, the raster angles are set to 45° or 135° for achieving a high-strength polymer AM-ed component. This research focuses on exploring the inherited anisotropic behaviors; thus, a combination of raster angles was chosen for a bilayer rectangular sheet of size 25 × 50 mm. In addition, the layer thickness and printing speed were designed at different levels for analyzing the intercorrelated morphing effects. The varied AM-ed processing variables are shown in Table 2.

Temperature was used as the stimulation method for this project. Bilayer-printed structures were immersed in hot water of 80 °C after printing, and the hot water was left to cool down to room temperature so that the after-morphed shape could be maintained. After the water cooled down to room temperature, the samples were removed from the water bath. The deformed bilayer structures were then measured for **Bending Angles** and **Curvatures** which are the two indicators for the **morphing performances**. As a measure of curvature, the curvature normal was obtained from the surface topology that is perpendicular to the axis of bending. Bending Angle describes the axis of inclination of the bending.

### Morphing Performance Measurements

**Figure 4:**
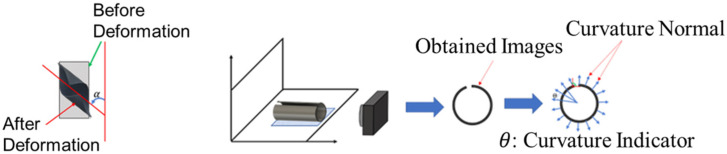
Bending Angle Measuring and Curvature Measurement Workflow.

The deformed bilayer structures were laid flat, ***Bending Angles*** were measured based on the contacting points of the freeform structures and the flat surface. The inclination angle (α) was recorded as the first morphing indicator. These angles for each sample were measured in a counterclockwise direction, as shown in the figure below. Curvature normal were obtained from the pictures of the deformed bilayer structures. The pictures were captured at an angle that was perpendicular to the bending orientation. The obtained shapes were then processed for the curvature normal. The ***curvature indicator*** was obtained from the consecutive curvature normal. These curvature indicators were then used to develop a machine learning model that can quantitatively predict the morphing responses when the AM-ed parameters are taken as inputs. The curvature indicators’ measurement process is shown in Figure 4.

## 4. Methodology

The collected data which represent the outputs, Bending Angle ($y_{2}$), and curvature normal ($y_{1}$) were then analyzed using regression analysis and artificial neural network model, respectively. The Gaussian process regression model was first adopted to describe the intercorrelated relationship between the morphing prediction controllers (thickness, printing pattern, and speed) and the Bending Angle. Gaussian process regression (GPR) can model a nonlinear relationship between variables. It is a type of Bayesian regression that uses a Gaussian distribution to model the uncertainty of the predicted values. GPR is well-suited for problems where the data are too small to fit a traditional linear model, or where the relationships between the variables are too complex to be captured by a linear model. Additionally, GPR provides a measure of uncertainty for the predicted values, which is important for making informed decisions. The regression function modeled by a multivariate gaussian is given as:

P(f|Χ)=N(f|μ,Κ)

where $Χ=\left[x_{1},x_{2},\;\ldots ,\;x_{n}\right]$; $f=\left(f\left(x_{1}\right),\;\ldots f\left(x_{n}\right)\right);\mu =\left[m\left(x_{1},\;\ldots ,m\left(x_{n}\right)\right)\right]$ and $K_{ij}=k\left(x_{i},x_{j}\right).\;Χ$ are the observed datapoints, *m* represents the mean function, and k represents a positive definite kernel function. With null observations, the mean function is defaulted to be m (Χ) = 0, provided the data are often normalized to a zero mean. The Gaussian process model is a distribution over functions whose shape (smoothness) is defined by Κ.

The predictive equations for GPR can be described as: $\bar{f_{*}}|X,y,X_{*}\sim N\left(\bar{f_{*}},cov\left(f_{*}\right)\right)$ where $\bar{f_{*}}≜E\left\{\bar{f_{*}}|X,y,X_{*}\right\}={Κ}_{*}^{Τ}{\left[Κ+\sigma_{n}^{2}I\right]}^{-1}y$, $cov\left(f_{*}\right)={Κ}_{**}-{Κ}_{*}^{Τ}{\left[Κ+\sigma_{n}^{2}I\right]}^{-1}{Κ}_{*}$.

It can be noted that in the variance function cov($f_{*}$) does not depend on the observed output y but only on the inputs X and ${Χ}_{*}\;$ [40]. The basic Gaussian distribution functions are usually chosen to be orthogonal, so that the model can be efficiently computed. The parameters of the Gaussian process are the mean and covariance of the basic functions.

A Levenberg–Marquardt training algorithm [41] was applied to the neural network model with 15 hidden layers for predicting the quantitative morphing degrees—curvature. A total of 15% of the data were set for validation and 15% of the data were used for testing, the remaining data were used for training. The Levenberg–Marquardt algorithm blends the steepest descent method and the Gauss–Newton algorithm. It performed well in this proposed research, since it can converge well even if the error surface is much more complex than the quadratic situation. Sum square error (SSE) is defined as $E\left(x,w\right)=\frac{1}{2}\underset{p}{\sum }\underset{m}{\sum }e_{p,m}{}^{2}\;$ to evaluate the training process, x and w represents the input and weight vectors, $e_{p,m}$ indicates the training error which can be defined as $e_{p,m}=d_{p,m}-o_{p,m}$. d and o are the desired and actual output vector—curvature.

## 5. Results

**Figure 5:**
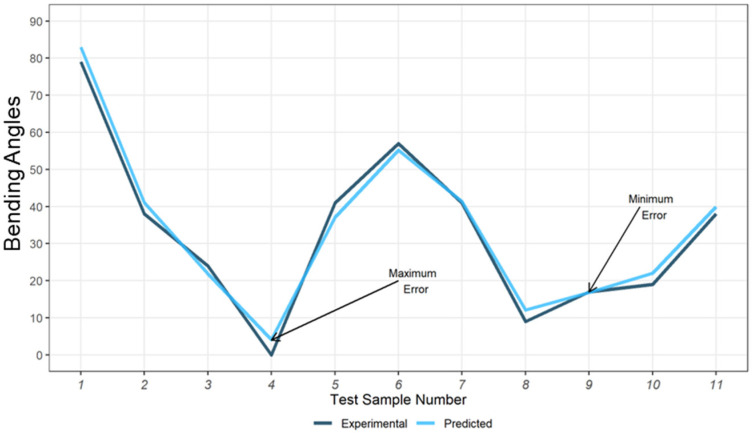
Morphing Bending Angle Prediction and Experimental Deviation.

Several bilayer structures with two layers in different printing directions, print speed, and layer thickness were fabricated, keeping other parameters constant, and the same shape-morphing stimulant temperature was applied. Based on the Bending Angle prediction model, the experimental validation is presented in Figure 5. The printing parameters for the test samples are shown in Table 3. The corresponding samples were tested under the same conditions as the training samples. The dark line represents the experimental results, and the blue line represents the result from our prediction model.

In Figure 5, the minimum and maximum prediction errors are indicated on the graph. The bending angle prediction model based on the three controlling factors (layer thickness, printing pattern, and speed) also presented a $R^{2}=0.9987$ which verifies the effectiveness and accuracy of the model.

**Figure 6:**
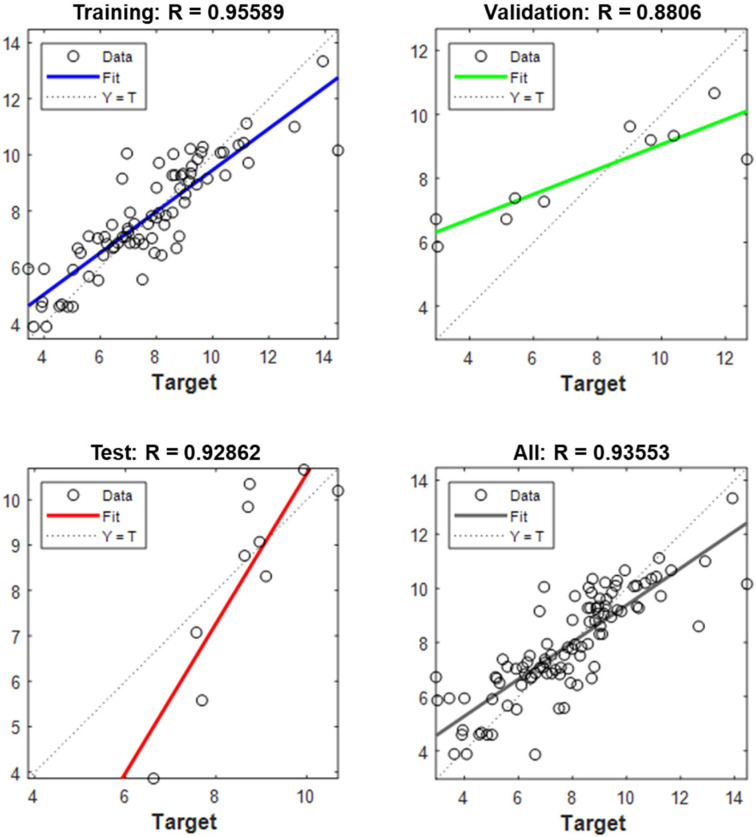
Neural Network Model $R^{2}$ values for test, training, validation, and all data.

Curvature, the other indicator of the self-morphing performance, has been predicted using the artificial neural network model, and the $R^{2}$ are presented in Figure 6 based on the training, validation, and the test data. The neural network framework is defined by the training data. The number of layers and neurons are determined by the number of data points in the training set. The input layer will be connected to the output layer, and the hidden layers will be connected to the input layer and the output layer. The neurons in the hidden layers will be connected to each other. The weights of the connections will be determined by the training data. The overall $R^{2}=0.93$ which also shows that the neural network model provides the sounding results.

The curvature analysis is also verified through the experimental validation, the comparison between the prediction and the experimental are shown in Table 4.

As we can see, the predicted values for curvature are consistent with the corresponding experiments, proving the validity of the NN model.

With the proposed 4D-printing strategy, various complex shapes can be created through the self-morphing production cycle based on simple AM-ed geometries. These 4D-printed shapes can reduce material usage, specifically on the support materials, and the production time. Based on the two prediction models, we can control the specific morphing shapes and also guide the initial AM-ed flat structure designs. In addition, the “flat structure” after the stimulation condition can also be introduced through the anisotropic-canceling behavior at the bi-layer model, as shown in the last column of Table 5. Other as-desired complex three-dimensional surface models are provided in the first row in the table, and the associated 3D prints are shown in the second row. All blue-colored volumes are the supports, which need to be removed later. The specific design of the AM-ed flat model and the processing conditions are presented in the table. The after-morphing performance of these designs are presented in the last row.

For the structures shown in the table above, the production time, volume, and material usage can be significantly reduced, and the quantitative reduction analysis are presented in Table 6.

From our obtained results, the key distinctions between the proposed method [40] and the existing literatures are:

1. Integrate the morphing physics with the performance quantitatively;
2. Multi-functional/degrees of morphing demeanor can be performed through single-material FDM process;
3. Reverse design of the 3D-printed structure and appropriate process condition can be obtained when the desired after-morphing complex shapes are provided.

**Figure 7:**
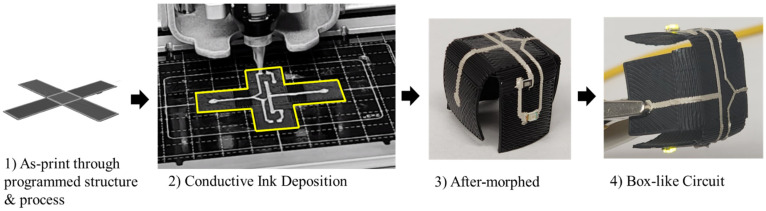
Box-like Circuit Design through the proposed programmable self-morphing method.

The programmed electronics are presented as a further application to present the capability of the proposed 4D-printing process. Current circuit printers are known to deposit conductive inks on a flat/planar surface. This hinders the design complexity of electronic devices. However, the conductive ink can be deposited to the pre-morphed shapes and activated through the environment to ultimately achieve complex geometries. Figure 7 presents a box-like circuit structure through the proposed methodology.

## 6. Conclusions and Discussion

Anisotropic behavior is exhibited when a material responds differently to stress depending on the direction of the applied force [5,9]. In the case of 3D printing, this can be seen when printing objects with a deposited path. When the filaments that are not aligned colinearly cross different layers, the object will have nonuniform properties. The 4D printing takes this a step further by using the anisotropic material behaviors that respond to environmental stimuli, such as heat or light [12,42,43]. This allows for objects that can change shape or even self-assemble based on the conditions around them.

Based on the proposed self-morphing method, the 4D printing performance can be predicted quantitatively by understanding the relationship between the printing parameters and the printing geometry. By adjusting the printing speed, layer thickness, and print pattern, the morphing behaviors of the printed object can be controlled. With these factors accounted for, the AM-ed flat structures and the associated processing conditions can be determined based on a set of given as-desired freeform surface models. Bending angle and curvature are selected as the morphing performance indicators, the $R^{2}$ achieves 99% and 93.5%, respectively. This hence justifies that the proposed 4D printing technique has a high repeatability and reproducibility to achieve the complex freeform geometries in a timely and robust manner.

## Figures and Tables

**Table 1:** Material Properties of Eco-ABS.

Properties	Values
Density	125 g/cm^3^
Thermal Expansion Coefficient	68 µm/m-K
Ultimate Tensile Strength	58 MPa
Glass Transition Temperature	78 °C

**Table 2:** AM-ed Processing Variables for Designing the Morphing Samples.

Parameters	Range
Path-layering Orientation	(0, 15, 30, 45, 60, 75, 90)
Layer Thickness	(0.1, 0.2, 0.3, 0.4)
Speed	(20, 25, 30, 40)

**Table 3:** Experimental Validation Samples Parameters.

Sample Number		1	2	3	4	5	6	7	8	9	10	11
Layer 1	Thickness $\left(t_{1}\right)$	0.3	0.3	0.3	0.3	0.3	0.4	0.4	0.3	0.4	0.3	0.3
	Print Angle $\left(\theta_{1}\right)$	45	0	10	65	35	80	25	25	10	35	5
	Print Speed $\left(s_{1}\right)$	25	25	25	25	25	25	25	25	25	25	25
Layer 2	Thickness $\left(t_{2}\right)$	0.1	0.1	0.1	0.1	0.1	0.2	0.2	0.2	0.2	0.2	0.2
	Print Angle $\left(\theta_{2}\right)$	10	45	65	85	50	35	40	70	65	60	40
	Print Speed $\left(s_{2}\right)$	27.5	27.5	27.5	27.5	27.5	27.5	27.5	27.5	27.5	27.5	27.5

**Table 4:** Curvature comparison based on the prediction model and the experimental analysis.

Sample Number		1	2	3	4	5
Layer 1	Thickness	0.4	0.4	0.3	0.3	0.3
	Print Angle	25	10	25	35	5
	Print Speed	25	25	25	25	25
Layer 2	Layer Thickness	0.2	0.2	0.2	0.2	0.2
	Print Angle	40	65	70	60	40
	Print Speed	27.5	27.5	27.5	27.5	27.5
Measured Curvature		6.91	12.97	6.82	6.74	5.35
Predicted Curvature		7.35	11.81	6.81	5.96	5.71
Deviation %		6.32	8.94	0.15	11.61	6.8

**Table 5:** AM-ed Structures for Self-morphing Applications in comparison to Traditional 3D Prints.

	Single-Fold Petals	Two-Fold Petals	Twisted Flower	Double-Sided Fold	Flat Structure
Desired shape	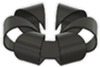	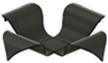	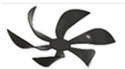	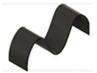	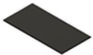
3D Printable Data (Blue – Supports; Red – 3D Model)	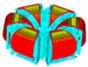	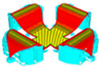	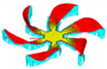	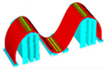	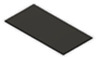
4D Print Design	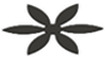	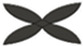	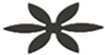	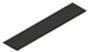	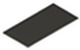
4D Print Process	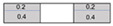	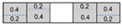	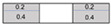	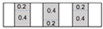	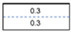
	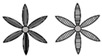	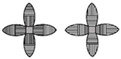	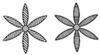	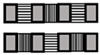	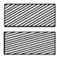
After-Morphed	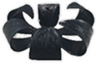	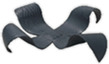	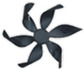	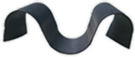	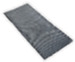

**Table 6:** Production time, volume, material usage reduction from the presented 4D-printing method.

		Print Time (min)	$\mathrm{Volume}\;(mm^{3})$	Material Usage (g)
Two-Fold Petals	3D	44.00	28,514.40	3.00
	4D	10.00	6000.00	1.00
	%Red	−77.27%	−78.96%	−66.67%
Double Sided Fold	3D	77.00	37,135.60	6.00
	4D	8.00	1200.00	1.00
	%Red	−89.61%	−96.77%	−83.33%
Single Fold Petals	3D	73.00	27,922.50	6.00
	4D	10.00	4399.44	1.00
	%Red	−86.30%	−84.24%	−83.33%
Twisted Flower	3D	57.00	70,599.29	4.00
	4D	10.00	4399.44	1.00
	%Red	−82.46%	−93.77%	−75.00%

## Data Availability

Available upon request.
